# Microclimate feedbacks sustain power law clustering of encroaching coastal woody vegetation

**DOI:** 10.1038/s42003-021-02274-z

**Published:** 2021-06-16

**Authors:** Heng Huang, Philip A. Tuley, Chengyi Tu, Julie C. Zinnert, Ignacio Rodriguez-Iturbe, Paolo D’Odorico

**Affiliations:** 1 Department of Environmental Science, Policy, and Management, University of California, Berkeley, CA USA; 2 Department of Biology, Virginia Commonwealth University, Richmond, VA USA; 3 School of Ecology and Environmental Science, Yunnan University, Kunming, Yunnan China; 4 Department of Ocean Engineering, Texas A&M University, TX USA

**Keywords:** Ecosystem ecology, Climate-change ecology

## Abstract

The spatial pattern of vegetation patchiness may follow universal characteristic rules when the system is close to critical transitions between alternative states, which improves the anticipation of ecosystem-level state changes which are currently difficult to detect in real systems. However, the spatial patterning of vegetation patches in temperature-driven ecosystems have not been investigated yet. Here, using high-resolution imagery from 1972 to 2013 and a stochastic cellular automata model, we show that in a North American coastal ecosystem where woody plant encroachment has been happening, the size distribution of woody patches follows a power law when the system approaches a critical transition, which is sustained by the local positive feedbacks between vegetation and the surrounding microclimate. Therefore, the observed power law distribution of woody vegetation patchiness may be suggestive of critical transitions associated with temperature-driven woody plant encroachment in coastal and potentially other ecosystems.

## Abstract (web summary)

Huang et al. use satellite imagery spanning over 40 years to investigate the spatial patterning of vegetation patches in a North American coastal ecosystem. They find that woody plant encroachment follows a power law when approaching critical transition points, which may inform future ecological monitoring of coastal systems.

## Introduction

Ecosystem state changes including abrupt shifts in vegetation type have been extensively documented in a variety of ecosystems across the globe, which often arise from internal feedbacks^1,2^. For example, the poleward expansion of cold-sensitive woody species into adjacent grasslands has been observed in many ecosystems worldwide, ranging from arctic tundras to desert and coastal grasslands^3–8^. This widespread shift in vegetation dominance has profound impacts on carbon sequestration, ecosystem productivity, and resilience^9–11^. Woody plant encroachment may result from a number of factors, including climate warming, increasing atmospheric CO_2_, nitrogen deposition, and land use change^11–14^. In regions where freezing stress has historically limited the expansion of woody plants, global or regional warming may reduce the frequency of extreme cold events and the associated freeze-induced mortality of woody plants^7,8,15^. This phenomenon can be enhanced by local-scale vegetation-microclimate feedbacks, which can also play an important role in driving the range shifts of many cold-sensitive woody species by altering the near-surface energy balance, thereby reducing cold stress exposure^7,16,17^. Specifically, woody canopies can absorb part of the nighttime long-wave radiation from the ground surface and then reflect or reradiate it back to the ground, thus reducing radiative cooling and creating a warmer microclimate compared to adjacent open canopy areas and grasslands^16,18^. Because of this positive feedback, a non-linear shift from one stable state with grass cover to another with woody plant dominance may occur in many cold-stressed ecotones worldwide when the minimum temperature increases above a critical threshold^8,16^.

Thus, important non-linearities may emerge in vegetation dynamics, including bifurcations and critical transitions. These ecosystem state changes are often highly irreversible and difficult to anticipate in real-world systems^19–21^. It is still unclear, however, how the spatial structure of vegetation changes in the course of such transitions and whether at the verge of a shift to the woodland state vegetation patterns exhibit the emergence of critical phenomena (scaling relations and power-law distributions) typical of systems approaching critical points^22–25^. For example, previous work has shown that the vegetation patches in some arid ecosystems including savannas may follow some characteristic distributions when the system is at the verge of a critical transition^20,26,27^. However, the spatial patterning of vegetation patches in ecosystems primarily controlled by other factors such as temperature has not been investigated yet. Previous work suggests that temporal dynamics of vegetation patterns may provide robust evidence of ecosystem regime shifts^28^, although recent modeling studies questioned the universality of indicators of critical transitions based on patch size distribution^29^. Therefore, exploring the temporal changes in vegetation patterning and the underlying temperature-associated mechanisms is important for understanding and predicting vegetation cover change and ecosystem resilience under global climate change in these temperature-driven ecosystems.

**Fig. 1:**
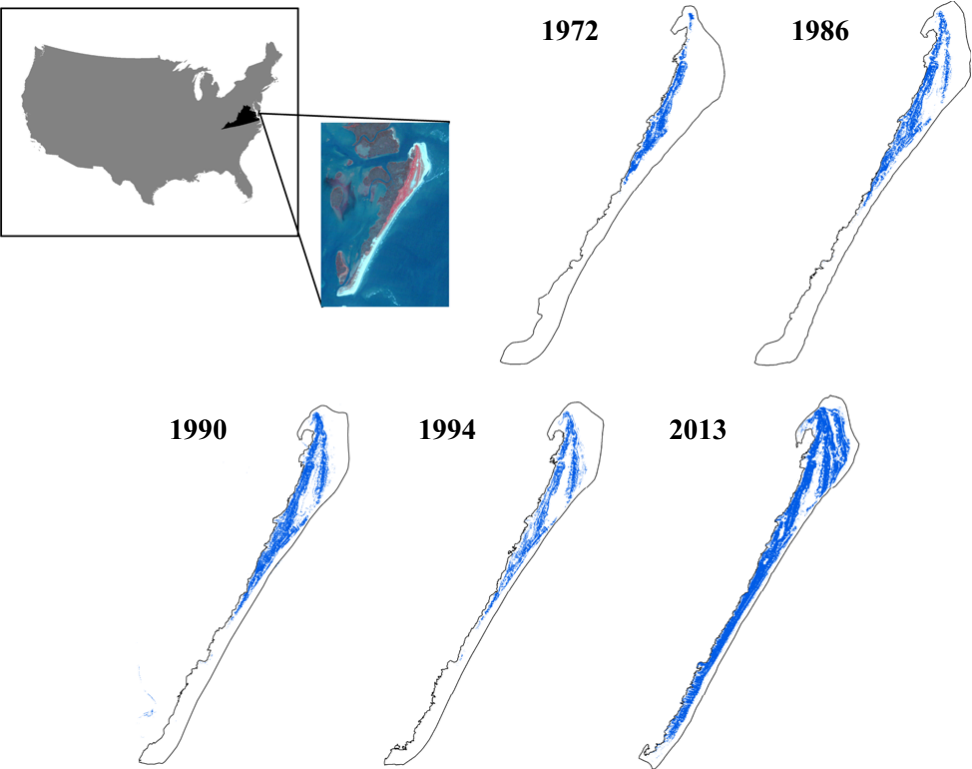
Changes in shrub cover on Hog Island, Virginia, USA from 1972 to 2013. The blue pixels indicate shrub patches and the upland outline (excluding marsh) is denoted each year in black. The shrub cover is 0.103 (1972), 0.150 (1986), 0.155 (1990), 0.154 (1994), and 0.403 (2013), respectively. The image inset is Landsat imagery courtesy of the U.S. Geological Survey.

Recent technological advances in remote sensing offer new possibilities to critically examine the spatial patterning of vegetation patchiness in temperature-controlled ecosystems using high-resolution imagery data. Here we focus on a North American coastal ecosystem i.e., the barrier islands along the eastern shore of Virginia, USA, where the woody species, *Morella cerifera* L., has encroached into adjacent grasslands over the last century^30^. A nearly 40% increase in *M. cerifera* cover was observed across these islands. For instance, on Hog Island (Virginia) the shrub cover has reached about 50% (Fig. 1)^7,30^. Recent work has documented the occurrence of positive feedback between the establishment of *M. cerifera* shrubs and microclimate^31,32^. Our parametrized model shows the emergence of a critical transition as a result of this positive feedback^7^, suggesting that the observed shift in plant cover is associated with a bifurcation in the underlying plant community dynamics. Here we investigate the spatiotemporal dynamics of woody plant encroachment and document how, as they approach the critical point, spatial patterns of vegetation exhibit power-law distributions of patch size.

We use a combination of high-resolution imagery data from 1972 to 2013 and a process-based modeling framework to examine the size (area) distribution of woody patches and its dependence on background climate conditions. Specifically, we tested (i) whether the spatial patterning can be described by a power law in some specific years; (ii) the extent to which these empirical patterns can be explained by our physical understanding of the underlying processes through a mechanistic model of vegetation dynamics capturing the complexity of the system; and (iii) whether the spatial patterns of woody vegetation change over time.

## Results and discussion

**Fig. 2:**
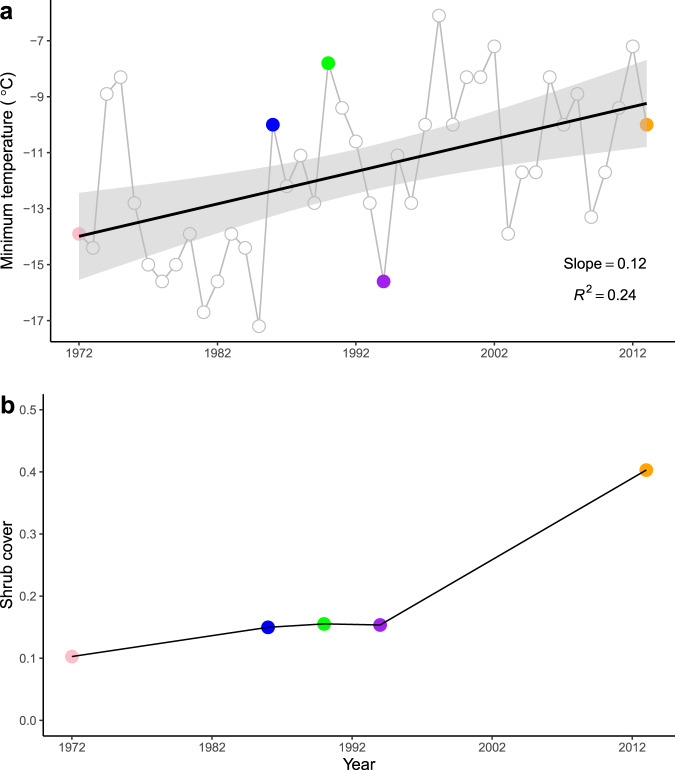
Long-term changes in minimum temperature and shrub cover. **a** Changes in annual lowest minimum temperature in Painter station, Virginia from 1972 to 2013. **b** Shrub cover change on Hog Island, Virginia from 1972 to 2013. The shaded area represents the 95% confidence interval range.

The long-term historical climate data from a nearby NOAA meteorological station in Painter, Virginia shows that the annual minimum temperature significantly increased from 1972 to 2013 on Virginia barrier islands (Fig. 2a; slope =  0.12, *P* < 0.001). This long-term climate warming is consistent with the observed expansion of the cold-sensitive *M. cerifera* shrub across Hog Island (Fig. 1) and the overall increase in shrub cover over this period (Fig. 2b).

**Fig. 3:**
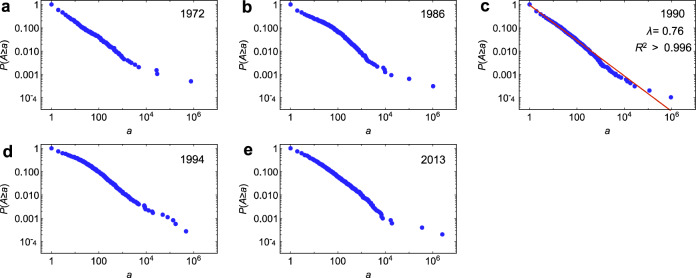
The observed size distribution of woody patches on Hog Island based on imagery data. The size distributions were shown for the following years: 1972 (**a**), 1986 (**b**), 1990 (**c**), 1994 (**d**) and 2013 (**e**). The size distribution follows a power law in 1990.

The results from imagery data show that the size distribution of shrub patches on Hog Island varied from 1972 to 2013. Around the year 1990, the shrub patch size distribution followed a power law, *P*[*A* ≥ *a*] ~ *a*^−*λ*^ (*λ* = 0.76, *R*^2^ > 0.99; Fig. 3c), while both in the previous and in the subsequent years it deviated from a power law (Fig. 3a, b, d, e). As expected, the number of larger patches and their size increased between 1972 and 2013 in the course of the encroachment process. In the year 1990, the distribution of shrub patch sizes followed a power law. The year 1990 preceded the relatively rapid expansion of *M. cerifera* across the island. The emergence of power-law distributions suggests that the shift in species dominance may be associated with a phase transition. Interestingly, climate data show that minimum temperature decreased from 1990 to 1994, which results in a slight decrease in shrub cover over this period (Fig. 2). This may explain why shrub patch size distribution in 1994 deviated from power law compared to the case of 1990.

Previous work has investigated the dynamics of shrub expansion in the time domain and shown how positive feedbacks between shrubs and microclimate may induce critical transitions^16^. The occurrence of such feedbacks is supported by empirical evidence of nocturnal warming in the presence of *M. cerifera* and the cold sensitivity of this species, which undergoes a significant decline in xylem conductivity as the temperature drops below −15 °C^7^. Interestingly, the last five decades have seen a consistent warming trend in the Eastern shore of Virginia, which has reduced *M. cerifera*’s exposure to cold (*T* < −15 °C) events (Fig. 2a). To explain the processes underlying these critical phenomena and the occurrence of a phase transition in the spatiotemporal dynamics of woody plant encroachment, we developed a stochastic cellular automata model that accounts for the vegetation-microclimate feedbacks, expressing the local facilitation by adjacent woody canopies on the transition rate of grasses to shrubs. We used this model to examine whether the observed power law in patch size distribution may emerge from positive vegetation-microclimate feedbacks (see “Methods” for details). The model was parameterized using experimental data from field and laboratory measurements^7^ or estimated empirically to provide realistic representations of the driving processes.

**Fig. 4:**
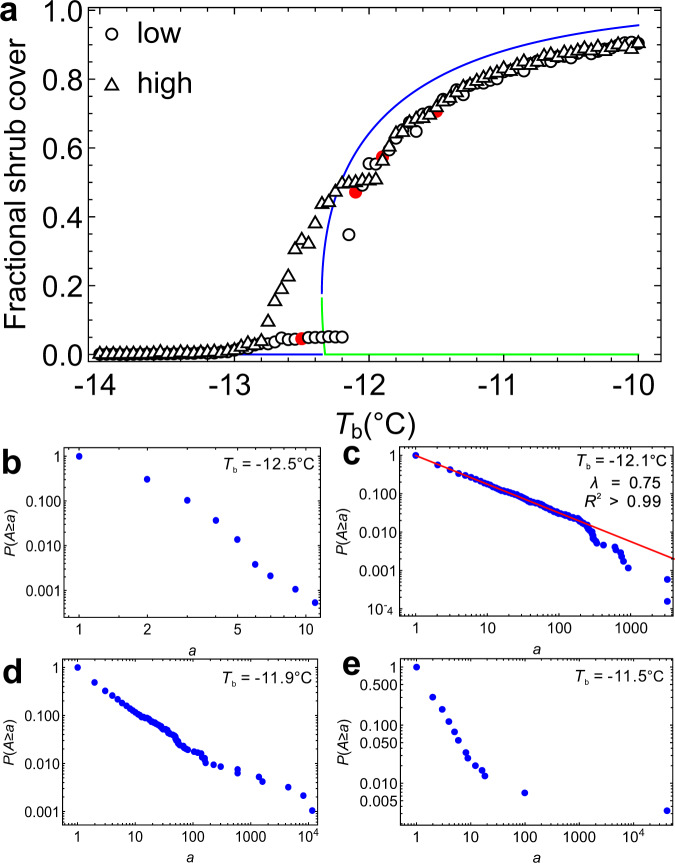
The ecosystem stable states (indicated by shrub cover) under varying background minimum temperature (*T*_b_) conditions. We ran the model for both low initial shrub cover (0.05) and high initial shrub cover (0.5). **a** Bifurcation in the dynamics of woody vegetation on Hog Island. Both mean field analysis (line) and simulation results from the full spatial model are shown. Stable and unstable states are indicated by blue and green lines, respectively. The size distributions of woody patches under four *T*_b_ conditions are shown: −12.5 °C (**b**), −12.1 °C (**c**), −11.9 °C (**d**), and −11.5 °C (**e**). The size distribution follows a power law when *T*_b_ is close to −12.1 °C.

The modeling results show that ecosystem state may shift from grassland to shrubland with increasing background temperature *T*_b_ (Fig. 4a). Hysteresis was also found as evidenced by the different steady states attained by the model when ecosystem dynamics were simulated using a low (0.05) and a relatively high initial shrub cover (0.5). In addition, the patch-size distribution also changed depending on *T*_b_ conditions (Fig. 4b–e and Supplementary Fig. 1). We used the scenario with a low initial condition for exemplification since this is what happens in nature in the process of shrub encroachment. For example, the distribution deviated from a power law and was closer to an exponential distribution when *T*_b_ was lower than −12.1 °C (Fig. 4b). At these temperatures *M. cerifera* shrubs are likely to experience freeze-induced mortality via losses of xylem hydraulic conductivity^7^. Therefore, at these low temperatures most shrub patches were relatively small while the formation of large patches was prevented by freezing stress, as indicated by the exponential distribution. In contrast, we found that the size distribution can be overall described by a power law when *T*_b_ is close to −12.1 °C (Fig. 4c). At this temperature the warmer microclimate conditions that occur beneath woody canopies as the result of vegetation-microclimate feedbacks significantly reduce the cold-induced mortality of *M. cerifera* shrubs thereby triggering critical transitions from grassland to shrubland^7^. Interestingly, power-law distributions are detected in shrub patches only when the system is undergoing a critical state change. In fact, as *T*_b_ increased above −12 °C, the patch size distribution deviated from a power law (Fig. 4d, e). The existence of this sharp transition is explained by the fact that only a slight increase in minimum temperatures is needed to release shrubs from cold stress thereby allowing woody patches to continue to grow even without the warming effect of vegetation-microclimate feedbacks. This leads to an increased proportion of large patches (Fig. 4d, e). In the scenario with a high initial condition, we found similar changes in patch size distribution with increasing *T*_b_ but the power-law distribution occurs when *T*_b_ was slightly above −12 °C instead of −12.1 °C (Supplementary Fig. 2).

Overall, the spatial patterns of shrub patches obtained from the cellular automata model are consistent with the empirical observations from satellite data. For example, the shrub patches on Hog Island exhibited a power-law distribution in 1990 and deviated from a power law both before and after, suggesting that the emergence of these fractal properties is evidence of critical phenomena at the critical point. The −12.1 °C temperature at which the model predicts a power-law distribution of shrub patches likely corresponds to the limit for cold stress tolerance detected by physiological measurements in *M. cerifera*^7^. In particular, the scaling exponent *λ* estimated from the model (0.75) is very similar to the value 0.76 calculated from the 1990 imagery data.

To investigate the role played by the positive shrub-microclimate feedback in the emergence of critical behavior, we ran some simulations using a null model that does not account for the local warming effect in the neighborhood of shrub canopies (i.e., ▵*T*_max_ = 0). The null model results show different vegetation patterns at a given temperature compared to results from the model with positive feedbacks. Importantly, the ecosystem exhibited continuous transitions without bifurcations when positive feedback is absent, as shown by both full model simulations and mean-field analysis (Supplementary Fig. 3). Furthermore, we found that the size distribution followed a power law at a higher temperature −10.85 °C with respect to the critical temperature −12.1 °C found when the feedback is accounted for (Supplementary Fig. 3). In the null model such a power-law distribution is not induced by local interactions associated with positive feedbacks with the abiotic environment but is simply the result of a percolation effect. In fact, in the null model the power-law distribution is found only when the fractional shrub cover is close to 0.59, which corresponds to the percolation threshold for a square lattice^33,34^. According to percolation theory, random uncorrelated dispersal with a density of 0.59 should lead to power-law distribution in patch size even in the absence of local interactions or feedbacks in the system’s dynamics. These findings corroborate our proposition that the observed power law of vegetation patterning in 1990 is driven by the local-scale vegetation-microclimate feedbacks.

Previous efforts have demonstrated how positive vegetation-microclimate feedbacks are able to induce a non-linear transition from grassland to shrubland in the temporal dynamics of vegetation^7,8^. In the case of the spatiotemporal dynamics of shrub encroachment, both full model simulations and a mean-field analysis of our model’s dynamics show how these feedbacks may induce a bifurcation in the equilibrium states of woodland-grassland systems as a function of the background temperature conditions (Fig. 4a). Although the mean-field analysis does not take the spatial structure into account, it shows the non-linear behavior emerging in response to large-scale climate warming and local microclimate feedbacks. Specifically, the system exhibits a critical transition from a stable grassland state to a stable state dominated by shrub vegetation, which is consistent with the non-linear and abrupt change in shrub cover observed in the cellular automata model results. Notice that empirical data showed the emergence of power-law scaling in cluster size distribution when shrub cover was ~0.15, which is similar to the shrub cover at the bifurcation point where the critical transition occurs (Fig. 4a). This finding is also consistent with recent work showing that local positive feedback may lower the percolation threshold below 0.59 at which percolation in the null model occurs^29^. In this sense, the finding of a lower percolation threshold with respect to the null model may be interpreted as the evidence of positive feedback sustaining these woody plant encroachment dynamics. It is possible that the transition from grass- to the shrub-dominated state could be continuous without bifurcations and this transition may also exhibit power-law distributions at the percolation point. However, we argue that this is not the case in this system, given the much lower percolation threshold observed in the data and the critical transition emerging in model simulations (Fig. 4a). We should note that the results from mean-field analysis might not match exactly the cellular automata model results given that spatial structure may alter the dynamic behavior of the system.

Our empirical and modeling results suggest that power-law size distribution of woody patches emerges when the ecosystem approaches a critical transition from a grassland to a woody plant-dominated state. This power-law patterning arises from the local positive feedback between woody plants and microclimate where woody canopies create a local nocturnal warming effect^7,16^. Previous studies have found evidence of power-law scaling in patch size distributions in arid ecosystems where water is the major limiting factor^26,27,35–38^ and mudflat ecosystems^39^. In addition, there is a rich body of literature suggesting that critical slowing down (i.e., slower recovery rates following a small perturbation when ecosystems approach critical transitions) may serve as an early warning signal of regime shifts in ecosystems^38,40,41^. It is still unclear whether critical phenomena can also emerge in temperature-controlled shifts in plant community composition. Our study demonstrates that this characteristic spatial patterning of vegetation patchiness may be ubiquitous and may be suggestive of woody plant encroachment in coastal landscapes where shrub expansion is primarily limited by extreme low temperatures, although recent work indicates that the relationship between cluster-size distributions and ecosystem resilience may depend on the strength of local positive feedback^29^. We considered the case of woody vegetation encroachment on a coastal ecosystem, where the expansion of *M. cerifera*, which was historically limited by its cold sensitivity, has been recently enabled by warming trends (Fig. 1).

A previous study^26^ showed the emergence of a truncated power law at the verge of a critical transition from a vegetated state to a “desert” state in arid ecosystems. Our work shows that power-law distribution may be suggestive of critical transitions also in another type of widespread ecosystem state shift, namely, the transition from grassland to shrubland, which is here investigated in detail for a specific barrier island (Hog Island) along the eastern shore of Virginia. The power-law clustering observed in our study can be explained by the increased establishment of large patches when the system approximates the critical transition thereby contributing to the fat tails of power-law distributions. We note that the model presented in this study aims to mimic the spatial patterning of shrub patches observed on Hog Island only qualitatively. Thus, the modeling results are not expected to match the empirical patterns exactly because (i) the observed shrub cover may not be at a steady state; (ii) there might be other processes involved in the spatial patterning of shrub patches such as wind damage and storm surge, which are not taken into account in the model; and (iii) the lattice size used to run the cellular automata model was limited by the computational cost of the simulations.

Critical transitions are widespread phenomena in natural systems and are often difficult to diagnose. They often entail relatively abrupt and potentially irreversible changes in ecosystem structure and services^2,20^. Therefore, the characteristic distribution of vegetation clusters may serve as an indicator of a phase transition in plant dominance, which can be used to detect ecosystem response to regional and global environmental change. The local positive feedback between vegetation and microclimate appears to play a crucial role in driving large-scale shifts in species composition, vegetation spatial patterning, and ecosystem state changes.

## Methods

### Study site

Hog Island, Virginia (37°40’ N, 75°40’ W) is a stable barrier island of the Virginia Coast Reserve and is part of the Long-Term Ecological Research network. Expansion of *M. cerifera* into grassland has been documented since the 1970s on Hog Island and several other Virginia islands due to macro- and microclimatic warming (Supplementary Fig. 4)^7,30^. Recent studies have demonstrated that *M. cerifera* outcompetes grasses and forbs, resulting in ~7 m tall monospecific thickets that are light limited, preventing the growth of other species^31,32^. When gaps form in the canopy due to thicket age, shrubs re-establish in the understory, maintaining a shrub state. More detailed descriptions of the study site can be found in Huang et al.^7^.

### Satellite data processing and fragmentation analysis

To investigate the spatial patterns of *M. cerifera* on Hog Island, detailed maps of evergreen shrub cover were created using georectified aerial photography and hyperspectral imagery. Cloud-free aerial photography was obtained from USGS Earth Explorer for the following dates: 2 Dec 1972 (color infrared), 5 Jul 1986 (color infrared), 5 Jul 1990 (color infrared), and 20 Mar 1994 (RGB). In 2013, hyperspectral imagery was available for Hog Island, VA (48 band hyperspectral)^42^. A seamless mosaic was performed on the multiple images which made up the 1972 scene and imagery resolution ranged from 0.41 to 1 m^2^. Regions of interest (ROI) were selected in each year for shrub cover using the bands available in each image (ENVI 5.5.3, LH3 Harris Geospatial) based on geo-rectified aerial photography, field surveyed woody thickets of known age using a Trimble Geo-XT GPS unit^43^, and woody thicket sampling locations of known age^44^. Shrub thicket homogeneity and high leaf cover, and the evergreen leaf habit relative to the otherwise sparse grassland cover and diversity in the system create distinct boundaries that are ideal for interpretation of shrub cover^43^. After ROIs were selected, supervised classifications were performed using the maximum likelihood method. Accuracy assessments were performed for each classification (Supplementary Table 1).

The resulting shrub cover was exported to ArcGIS 10.7 (ESRI) and then exported to the program FRAGSTATS 4.2 for spatial pattern analysis^45^. We calculated the size (area, m^2^) of each shrub patch identified in satellite images. The study area, number of shrub patches, and range of patch size from 1972 to 2013 were summarized in Supplementary Table 2. We then fitted a power law using the NonlinearModelFit function in Mathematica 12.0 (Wolfram Research, USA) to the inverse cumulative distribution of shrub patch sizes, defined as *P*(*A* ≥ *a*), the probability of a cluster area *A* being greater than or equal to a given value *a*^35^. This approach has been demonstrated to provide more robust estimates of distribution parameters than other traditional approaches including fitting frequency distribution^25,46^.

### Cellular automata model

We developed a stochastic cellular automata model to account for the effects of positive vegetation-microclimate feedbacks on the spatiotemporal dynamics of *M. cerifera* and explain the emergence of power laws in shrub patch size distribution. The model simulations were performed on a lattice composed of 600 × 100 cells to mimic the shape of Hog Island which has a length of ~12 km and a maximum width of ~2 km. We considered two vegetation states, namely, shrubs (*S*) or grasses (*G*); each cell in the lattice had either *S* or *G* cover. We define *p*_*S*_ (*p*_*G*_) as the fraction of shrub (grass) cells in the whole lattice, and *q*_*S*|*G*_ (*q*_*G*|*S*_) the fraction of shrub (grass) cells in the von Neumann neighborhood of a grass (shrub) cell (the four nearest neighbors that share one edge with the focal cell).

We follow Kéfi et al.^36^ and model the transition probability of a *G* cell to a *S* cell (i.e., from grassland to shrubland) as follows

1 $${w}_{G\to S}=\alpha \left[\delta {p}_{S}+\left(1-\delta \right){q}_{S{{|}}G}\right]\left(1-{p}_{S}\right)$$

where *α* is the intrinsic growth rate of *M. cerifera* shrubs, *δ* is the fraction of shrub growth contributed globally by the presence of other shrubs in the domain and 1−*δ* is the fraction of shrub growth facilitated locally by the presence of shrubs in the von Neumann neighborhood. The term (1− *p*_*S*_) describes how the growth and establishment of shrub seedlings is constrained by limiting resources such as soil water and nutrients, light, and physical space.

The cold-induced mortality probability of shrubs in a *S* cell (i.e., transition from shrub to grass cover) depends on *T*_min_, the minimum temperatures within shrub thickets, and is expressed as

2 $${w}_{S\to G}=\beta f\left({T}_{{{\min }}}\right)$$

where *β* is the maximum mortality probability caused by freeze damage. *f*(*T*_min_) is a function describing how shrub mortality increases from 0 to the maximum with decreasing *T*_min_ (Supplementary Fig. 5)

3 $$f\left({T}_{{{\min }}}\right)=\frac{{\left|{T}_{{{\min }}}-{T}^{\ast }\right|}^{n}}{{\left|{h}_{{\rm{T}}}-{T}^{\ast }\right|}^{n}+{\left|{T}_{{{\min }}}-{T}^{\ast }\right|}^{n}}$$

where *h*_T_ is the temperature value at which shrub mortality rate is 50%, T^*^ is the temperature when shrubs start to experience freezing stress, and *n* is the parameter that determines the sharpness of the temperature response. The local warming effect, which is induced by the woody canopies through altering near-surface energy budget, results in a higher *T*_min_ compared to the background minimum temperature (*T*_b_) in adjacent open grassland^18,47^. The magnitude of this local warming effect is dependent on shrub density in the nearest neighborhood (*q*_*S*|*S*_ or 1−*q*_*G*|*S*_) and can be modeled through a linear function as

4 $${T}_{{{\min }}}={T}_{{\rm{b}}}+\triangle {T}_{{{\max }}}\left(1-{q}_{G{{|}}S}\right)$$

where △*T*_max_ is the maximum local warming effect.

We performed model simulations for different values of *T*_b_. For each of the given *T*_b_ scenarios, we ran the model until the patch cover reached a relatively stable state which is defined as the condition in which the change in the fractional cover of shrub patches between two consecutive time steps is smaller than 0.0001. After the stable state was reached, the number and size of shrub patches were calculated over 10-time steps and the mean patch size distributions for each *T*_b_ scenario were determined. The initial values of *p*_*S*_ and *q*_*G*|*S*_ were set to 0.05 for each simulation to reflect the fact that shrub cover increases from a relatively low value during shrub encroachment. We also ran model simulations using relatively high initial values of *p*_*S*_ and *q*_*G*|*S*_ (0.5) to examine the hysteresis i.e. the difference in the forward and backward responses of ecosystem dynamics to *T*_b_. The values of parameters were obtained from experimental data or estimated empirically based on shrub growth characteristics to reflect field conditions as in Huang et al.^7^. Therefore, *h*_T_ = −15 °C, T^*^ = 0 °C, *n* = 15, △*T*_max_ = 2 °C. The parameters *α* and *β* were set at 0.05 and 1, respectively, keeping the same *α*/*β* ratio as in the corresponding parameters in Huang et al.^7^. We assigned a much greater value to *β* compared to *α* to indicate that a full shrub canopy can collapse within a relatively shorter time frame (a few years) when high freeze stress occurs, compared to the time (several decades) required for shrub patch establishment. We used *δ* = 0.3 to reflect that shrub seedling establishment is more dependent on shrub density in the neighborhood. We should note that the modeling results are qualitatively insensitive to *δ*.

To further investigate the possible stable states of woodland-grassland systems under different background temperature conditions, we used mean-field analysis^36^. The mean-field analysis assumes that there is no spatial structure such that the global vegetation densities (*p*_S_ or *p*_G_) are equal to the local vegetation densities (*q*_S|G_ or *q*_G|S_). This simplification allows us to derive a single equation for woody plant growth

5 $${{\mathrm{d}}S}/{{\mathrm{d}}t}={p}_{G}{w}_{G\to S}-{p}_{S}{w}_{S\to G}=\left(1-{p}_{S}\right)\alpha {p}_{S}\left(1-{p}_{S}\right)-{p}_{S}\beta f\left({T}_{{\rm{min }}}\right)$$

The stable equilibrium points were determined at different *T*_b_ temperatures by setting ${{\mathrm{d}}S}/{{\mathrm{d}}t}=0$ in Eq. (5) and solving for it.

## Supplementary information

SUPPLEMENTAL MATERIAL

Reporting_Summary

## Data Availability

The climate data are available from NOAA (https://www.ncdc.noaa.gov). The imagery data are available from USGS Earth Explorer and the Virginia Coast Reserve Long-Term Ecological Research Project Data Publication (doi:10.6073/pasta/6a5cc305e93c2baf9283facee688c504).

## Code availability

The code for modeling analyses is available at: https://osf.io/xbwy5/.
