# Supplementary material for: Microclimate feedbacks sustain power law clustering of encroaching coastal woody vegetation
Source: Commun Biol. 2021 Jun 16;4:745. doi: 10.1038/s42003-021-02274-z (PMC8208994; doi:10.1038/s42003-021-02274-z)
Supplement: Supplementary file 1 — SUPPLEMENTAL MATERIAL [file 42003_2021_2274_MOESM1_ESM.pdf]

Supplementary Materials for

Microclimate feedbacks sustain power law clustering of encroaching coastal woody vegetation

Heng Huang[1]*, Philip A. Tuley[2], Chengyi Tu[1,3]*, Julie C. Zinnert[2], Ignacio Rodriguez-Iturbe[4], Paolo D'Odorico[1]*

[1]Department of Environmental Science, Policy, and Management, University of California, Berkeley, CA, USA

[2]Department of Biology, Virginia Commonwealth University, Richmond, VA, USA

[3]School of Ecology and Environmental Science, Yunnan University, Kunming, Yunnan, China

[4]Department of Ocean Engineering, Texas A&M University, TX, USA

*Correspondence to: henghuang@berkeley.edu (H.H.), chengyitu@berkeley.edu (C.T.) or paolododo@berkeley.edu (P.D.)

This PDF file includes:

Supplementary Figures 1 to 5

Supplementary Tables 1 to 2

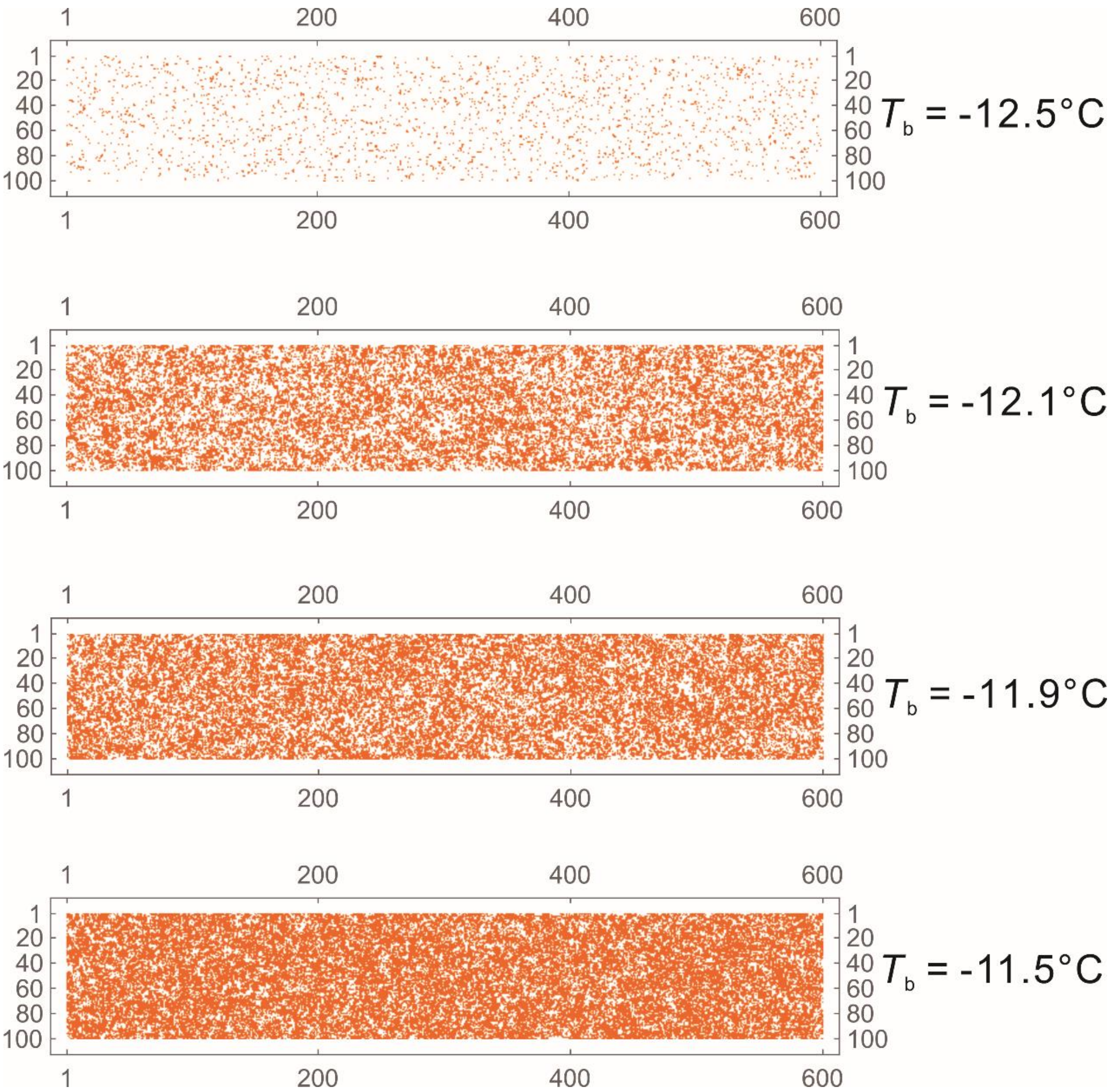

Supplementary Figure 1. The spatial configuration of shrubs from full model simulations at steady state with a low initial shrub cover (0.05).

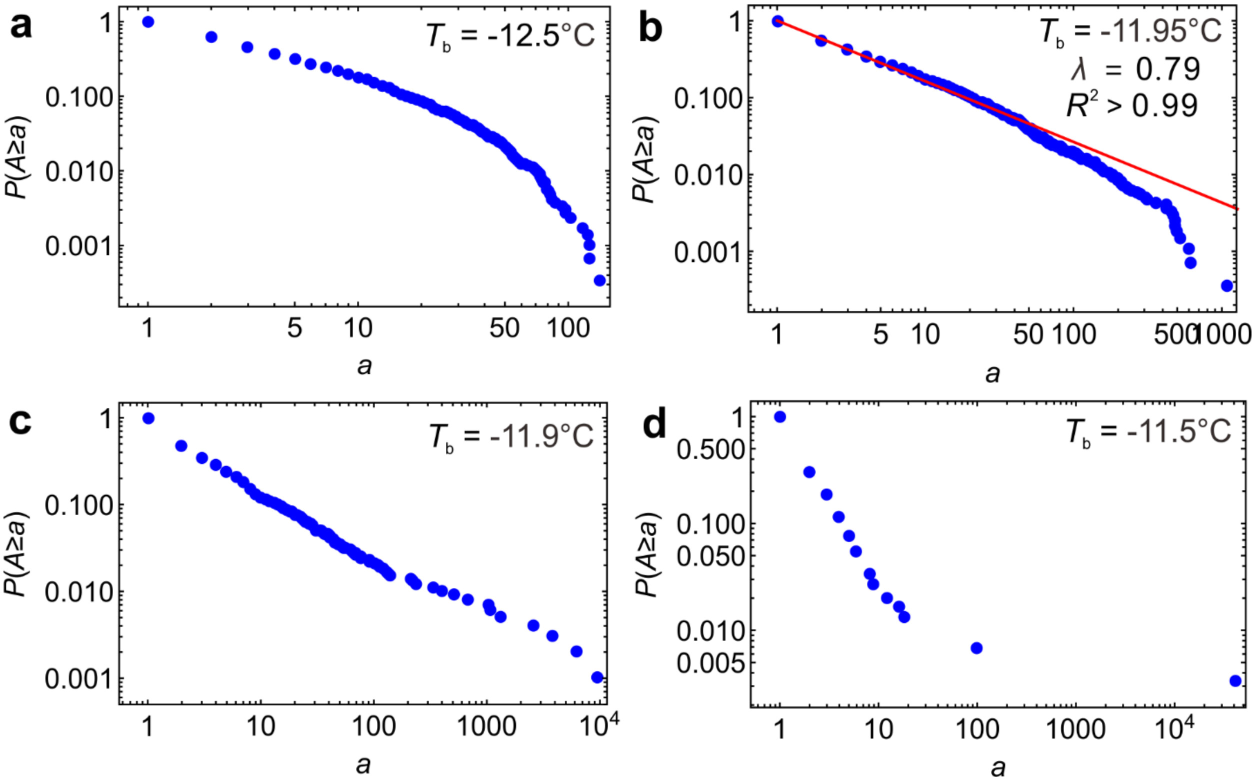

Supplementary Figure 2. The size distribution of woody patches under different background minimum temperature (T_b) conditions from model simulations with a high initial shrub cover (0.5).

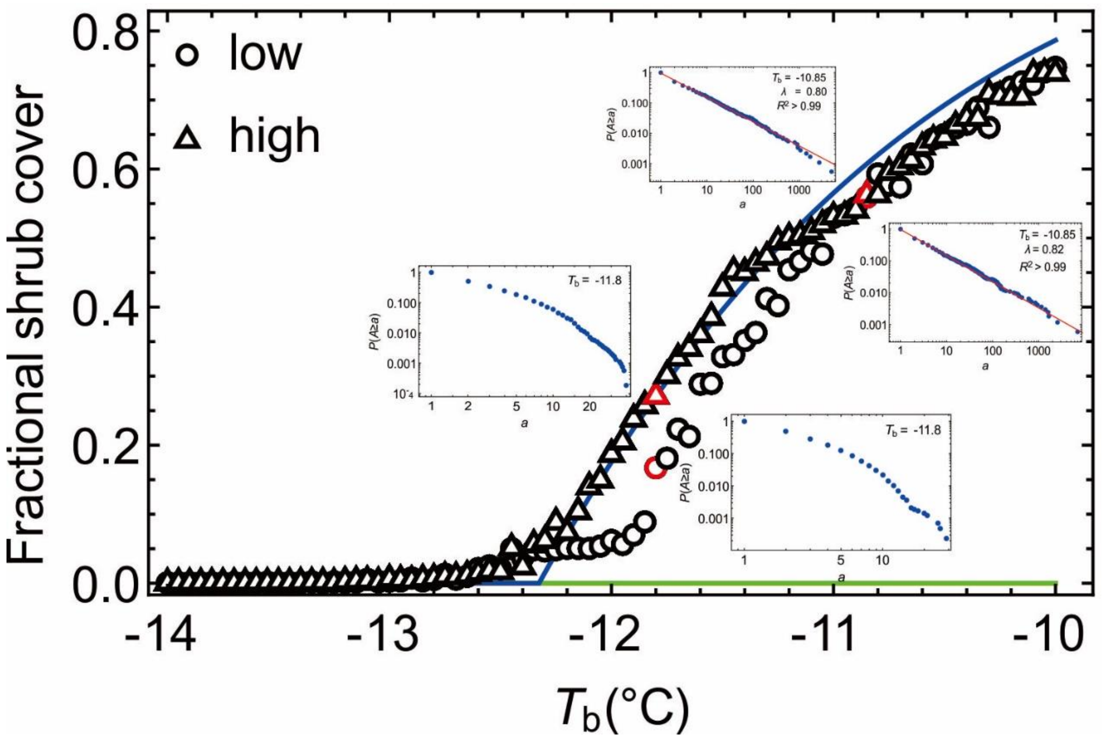

Supplementary Figure 3. The stable shrub cover and size distribution of woody patches under varying background minimum temperature (T_b) conditions when there are no positive vegetation-microclimate feedbacks ($\triangle T_{max}$ = 0°C). We ran the model for both low initial shrub cover (0.05) and high initial shrub cover (0.5), respectively. The blue line indicates the results from mean-field analysis and the points indicate the simulation results from the full spatial model. The patch size distributions at two temperatures (−11.8°C and −10.85°C) are shown for exemplification.

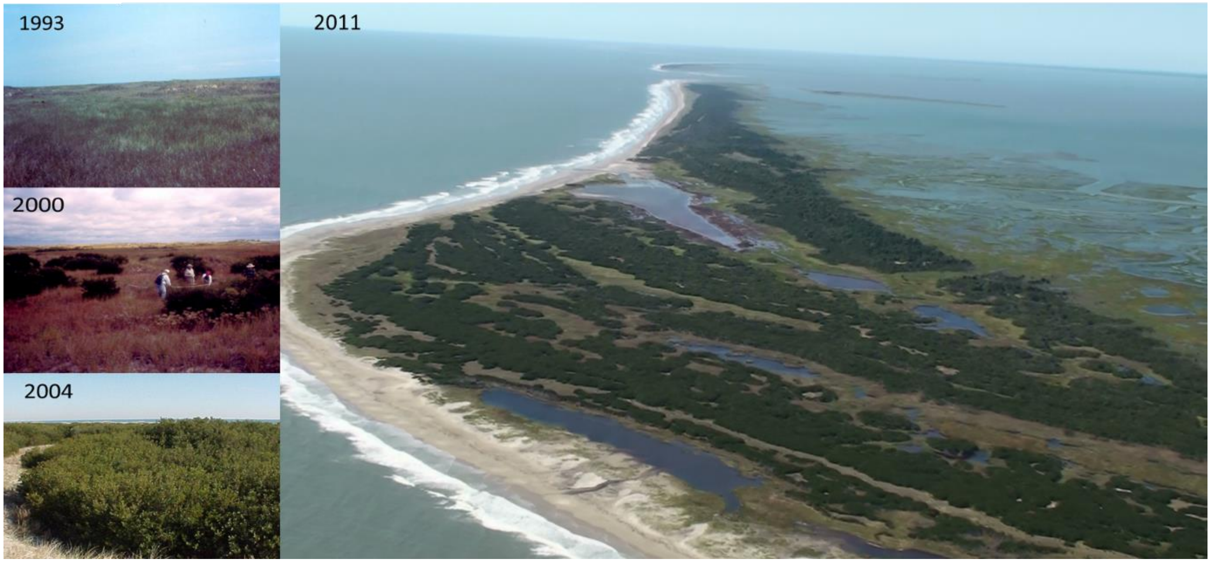

Supplementary Figure 4. Photos of shrub encroachment into grassland on Hog Island, Virginia. Grassland converted to full shrub thicket within the same location in 11 years (left, photo credit: Donald Young). Aerial photo looking south along the island shows the patterning of shrubs across the landscape (right, photo credit: John Porter).

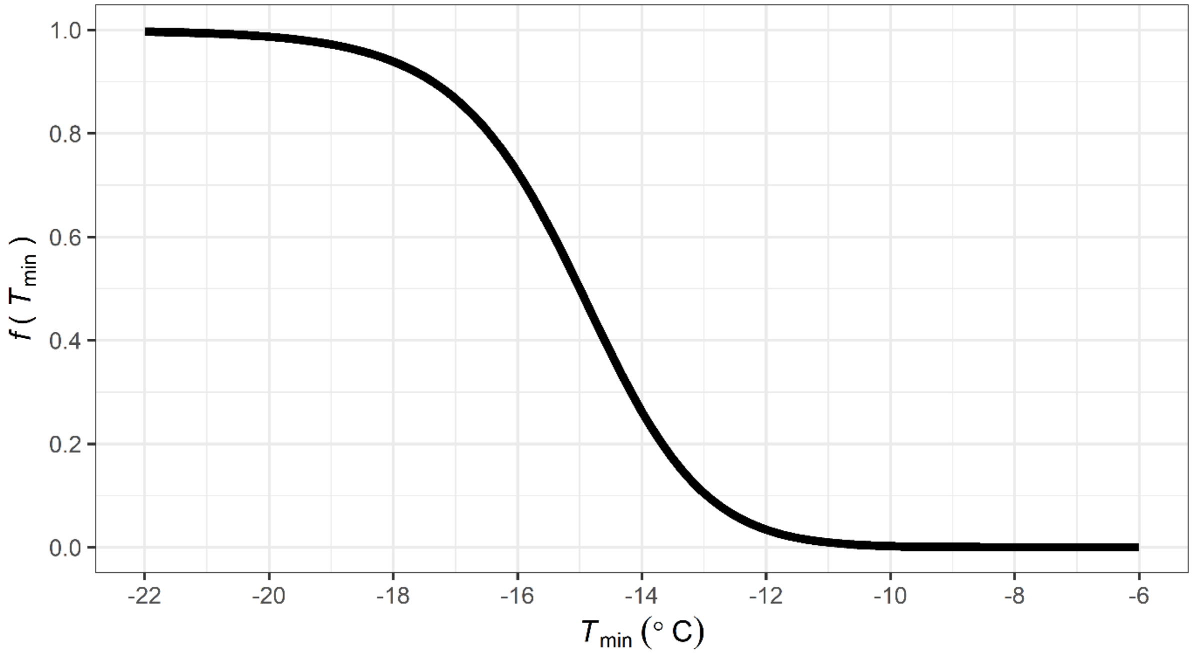

Supplementary Figure 5. The mortality rate of *M. cerifera* shrubs as a function of the minimum temperature under shrub canopies (T_{min}). See Eq. (3) for details.

Supplementary Table 1. Summary statistics of confusion matrices for each classification.

Year	% User Accuracy	% Producer Accuracy	% Overall Accuracy	Kappa
1972	100	100	100	100
1986	100	100	100	100
1990	100	95	97	94
1994	100	100	100	100
2013	100	85	91	83

Supplementary Table 2. Summary of shrub patches on Hog Island from 1972 to 2013.

Year	Size of study area (m^2)	Number of patches	Range of patch size (m^2)
1972	8268080	1932	1-746564
1986	8727000	3119	1-1049935
1990	8160420	9595	1-900027
1994	7809440	3551	1-476509
2013	7772550	4941	1-2509774
