# Supplementary material for: Microclimate feedbacks sustain power law clustering of encroaching coastal woody vegetation
Source: Commun Biol. 2021 Jun 16;4:745. doi: 10.1038/s42003-021-02274-z (PMC8208994; doi:10.1038/s42003-021-02274-z)
Supplement: Supplementary file 2 — Reporting_Summary [file 42003_2021_2274_MOESM2_ESM.pdf]

nature research

Corresponding author(s): Heng Huang

Last updated by author(s): Apr 27, 2021

Reporting Summary

Nature Research wishes to improve the reproducibility of the work that we publish. This form provides structure for consistency and transparency in reporting. For further information on Nature Research policies, see our Editorial Policies and the Editorial Policy Checklist.

Statistics

For all statistical analyses, confirm that the following items are present in the figure legend, table legend, main text, or Methods section.

n/a	Confirmed	
☒	☐	The exact sample size (n) for each experimental group/condition, given as a discrete number and unit of measurement
☒	☐	A statement on whether measurements were taken from distinct samples or whether the same sample was measured repeatedly
☐	☒	The statistical test(s) used AND whether they are one- or two-sided<br>*Only common tests should be described solely by name; describe more complex techniques in the Methods section.*
☒	☐	A description of all covariates tested
☒	☐	A description of any assumptions or corrections, such as tests of normality and adjustment for multiple comparisons
☒	☐	A full description of the statistical parameters including central tendency (e.g. means) or other basic estimates (e.g. regression coefficient) AND variation (e.g. standard deviation) or associated estimates of uncertainty (e.g. confidence intervals)
☐	☒	For null hypothesis testing, the test statistic (e.g. F, t, r) with confidence intervals, effect sizes, degrees of freedom and P value noted<br>*Give P values as exact values whenever suitable.*
☒	☐	For Bayesian analysis, information on the choice of priors and Markov chain Monte Carlo settings
☒	☐	For hierarchical and complex designs, identification of the appropriate level for tests and full reporting of outcomes
☒	☐	Estimates of effect sizes (e.g. Cohen's d, Pearson's r), indicating how they were calculated

Our web collection on statistics for biologists contains articles on many of the points above.

Software and code

Policy information about availability of computer code

Data collection	Data were acquired from NOAA, USGS Earth Explorer and the Virginia Coast Reserve Long-Term Ecological Research Project Data Publication. Details were given in the data availability statement.
Data analysis	The spatial analyses were done using ArcGIS (version 10.7), ENVI (version 5.5.3), and FRAGSTATS (version 4.2). R software (version 3.6.1) and Mathematica (version 12.1) were used for statistical analyses.

For manuscripts utilizing custom algorithms or software that are central to the research but not yet described in published literature, software must be made available to editors and reviewers. We strongly encourage code deposition in a community repository (e.g. GitHub). See the Nature Research guidelines for submitting code & software for further information.

Data

Policy information about availability of data

All manuscripts must include a data availability statement. This statement should provide the following information, where applicable:

- Accession codes, unique identifiers, or web links for publicly available datasets
- A list of figures that have associated raw data
- A description of any restrictions on data availability

The climate data are available from NOAA (https://www.ncdc.noaa.gov). The imagery data are available from USGS Earth Explorer and the Virginia Coast Reserve Long-Term Ecological Research Project Data Publication (doi:10.6073/pasta/6a5cc305e93c2baf9283facee688c504).

Field-specific reporting

Please select the one below that is the best fit for your research. If you are not sure, read the appropriate sections before making your selection.

☐ Life sciences ☐ Behavioural & social sciences ☒ Ecological, evolutionary & environmental sciences

For a reference copy of the document with all sections, see nature.com/documents/nr-reporting-summary-flat.pdf

Ecological, evolutionary & environmental sciences study design

All studies must disclose on these points even when the disclosure is negative.

Study description	In this study, we focus on a North American coastal ecosystem and integrate high-resolution imagery data with a novel stochastic cellular automata model to investigate the spatial patterning of woody patches and the underlying mechanisms driving these patterns. Classifications were performed for all images using the maximum likelihood method to distinguish shrubs from other land cover types. We then calculated the size (area) of each shrub patch identified in satellite images.
Research sample	The entire imagery data for Hog Island, Virginia, USA were used for analyses and no samples were used in this study.
Sampling strategy	No sampling strategy was used in this study.
Data collection	Data were collected by NOAA, USGS, and USACE Engineer Research and Development Center.
Timing and spatial scale	The imagery data for Hog Island, Virginia, USA were acquired for the following dates: 2 Dec 1972, 5 Jul 1986, 5 Jul 1990, 20 Mar 1994, and 26 May 2013.
Data exclusions	No data were excluded from the analyses.
Reproducibility	The code is publicly available to ensure reproducibility.
Randomization	This study analyzed the spatial patterning of vegetation using high-resolution imagery data and did not involve experimental randomization.
Blinding	Blinding was not relevant to this study.

Did the study involve field work? ☐ Yes ☒ No

Reporting for specific materials, systems and methods

We require information from authors about some types of materials, experimental systems and methods used in many studies. Here, indicate whether each material, system or method listed is relevant to your study. If you are not sure if a list item applies to your research, read the appropriate section before selecting a response.

Materials & experimental systems

n/a	Involved in the study	
☒	☐	Antibodies
☒	☐	Eukaryotic cell lines
☒	☐	Palaeontology and archaeology
☒	☐	Animals and other organisms
☒	☐	Human research participants
☒	☐	Clinical data
☒	☐	Dual use research of concern

Methods

n/a	Involved in the study	
☒	☐	ChIP-seq
☒	☐	Flow cytometry
☒	☐	MRI-based neuroimaging
